# Graphene Oxide (GO) Impregnation of Polyamide-Based Composites Enhances Thermal Conductivity After Selective Laser Sintering

**DOI:** 10.3390/nano16030170

**Published:** 2026-01-27

**Authors:** Viktoria A. Koshlakova, Andrey A. Stepashkin, Valter Maurino, Dmitry S. Muratov

**Affiliations:** 1 Department of Functional Nanosystems and High-Temperature Materials, National University of Science and Technology “MISIS”, 119049 Moscow, Russia; vikakoshlakova@yandex.ru; 2 Center of Composite Materials, National University of Science and Technology “MISIS”, 119049 Moscow, Russia; a.stepashkin@yandex.ru; 3 Department of Chemistry, University of Turin, 10125 Turin, Italy; 4 UNITO-ITT JointLab, via Quarello 15/a, 10135 Turin, Italy

**Keywords:** thermoplastic composites, additive manufacturing, semicrystalline polymer, mechanical properties, toughness, graphene oxide, selective laser sintering

## Abstract

Selective laser sintering (SLS) is an additive manufacturing method that enables the creation of complex-shaped polymer-based structures with great control over the desired properties. In this study, polyamide 12 (PA12)–based powders containing 0.8 wt.% graphene oxide (GO), introduced via a wet-mixing impregnation method, were processed by selective laser sintering (SLS). Implementation of a double laser scanning strategy increased the tensile strength of the composites by 2.5% relative to pristine SLS-processed PA12 and enhanced the thermal conductivity to 0.74 W·m^−1^·K^−1^. The results indicate that the laser sintering process is an effective approach to produce low filler content polymer-matrix composites with enhanced thermal properties while preserving mechanical integrity and maintaining electrical insulation behavior.

## 1. Introduction

In recent years, polymer composite materials [1,2,3] have attracted increasing attention due to rapid advances in microelectronics, energy technologies, and biomedicine. Incorporating thermally conductive nanomaterials as fillers in polymer matrices enables the development of efficient thermal management systems [4], energy-storage devices [5], and flexible conductors [6]. Among the most promising nanofillers is graphene, owing to its outstanding mechanical strength, electrical conductivity, and exceptional thermal conductivity of 5000 W·m^−1^·K^−1^ [7]. However, the fabrication of polymer composites containing graphene remains costly and technically demanding, primarily due to challenges associated with achieving homogeneous dispersion and controlled interfacial interactions. Additive manufacturing offers a potential solution, as it enables the fabrication of complex three-dimensional structures with layer-by-layer deposition [8].

In Ref. [9], thermoplastic polyurethane (TPU) composites containing 5–45 wt.% graphene were fabricated by fused deposition modeling (FDM). Filaments were prepared by dissolving TPU in DMFA, mixing the solution with graphene powder, and subsequently extruding the blend. The resulting composites exhibited thermal conductivities of 1 W·m^−1^·K^−1^ (5 wt.% graphene) and 12.25 W·m^−1^·K^−1^ (45 wt.% graphene), compared with 0.25 W·m^−1^·K^−1^ for pure TPU. This significant enhancement was attributed to the anisotropic structure and preferential alignment of graphene along the print direction.

Selective laser sintering (SLS) is particularly promising for the fabrication of three-dimensional graphene-filled polymer composites owing to its geometric freedom, scalability, and ability to process powder-based feedstocks without the need for binders or support structures [10]. Polyamide 12 (PA12), the primary material used in SLS, is well suited for this process because of its wide processing window between melting and crystallization. In Ref. [11], PA12 was melt-mixed with flake graphite and graphene nanoplatelets at concentrations of 10–40 wt.% followed by hot pressing at 230 °C. Composites containing 40 wt.% filler exhibited a 2.4-fold increase in elastic modulus and thermal conductivities of 1.6 and 2.7 W·m^−1^·K^−1^ for nanoplatelet- and graphite-filled samples, respectively. Notably, the thermal conductivity at 10 wt.% nanoplatelets (0.5 W·m^−1^·K^−1^) was lower than that of pure PA12 (0.95 W·m^−1^·K^−1^), which the authors attributed to interfacial thermal resistance.

In Ref. [12], laser sintering was used to fabricate conductive PA12 composites coated with reduced graphene oxide (rGO). The rGO-coated PA12 powder was prepared by dispersing PA12 in an aqueous graphene oxide suspension in the presence of sodium chloride and hydrazine, where the later served as a reducing agent. Increasing rGO content (0–0.72 vol.%) led to a marked increase in electrical conductivity; composites containing 0.36 vol.% (≈1 wt.%) rGO reached 10.5 S·m^−1^ due to the formation of a continuous surface network. The printed composites with 0.36 wt.% rGO exhibited lower compressive modulus than pure PA12 (359 ± 30 vs. 473 ± 14 MPa), attributed to reduced interlayer adhesion. However, these composites demonstrated improved tensile modulus (221 ± 17 vs. 149 ± 26 MPa) and elongation (8.6 ± 1.2 vs. 8.7 ± 2.0%) relative to neat PA12.

Finally, both thermal and mechanical properties of PA12/Graphene fabricated by fused deposition modeling were addressed in [13]. While the highest obtained thermal conductivity at 10 wt.% of graphene nanoplatelets was shown to be higher than 1 W·m^−1^·K^−1^, it was achieved only along the printing direction. Compression molded samples has shown 51.4% lower thermal conductivity in comparison to printed along the measurement axis.

Despite these advances, laser sintering of graphene-based polymer composites remains challenging, as careful optimization of processing parameters is required to simultaneously achieve enhanced thermal performance, adequate mechanical strength, and reliable electrical insulation. In this work we show a comparison between hot pressed PA12/GO composites with two types of GO impregnation in SLS created structures: before and after the sintering. We also provide an optimized composite preparation procedure with enhanced thermal conductivity that does not impact mechanical properties and maintain electrical insulation and was successfully tested in modeled radiators for small-scale electronic devices.

## 2. Materials and Methods

Commercial polyamide 12 (PA12) powder (DuraForm^®^, 3D Systems, Rock Hill, SC, USA) was used as the polymer matrix. Graphene oxide (GO) supplied by Mineral Ltd (Mineral ltd., Alexandrov, Russia). was provided as an aqueous dispersion with a concentration of 15 mg·mL^−1^. PA12 powder was treated by the dispersion of GO for 30 min using a magnetic stirrer. The resulting mixture of PA12 with 0.8 wt.% GO (PA12/GO) was dried at 80 °C for 3 h followed by sieving through a 150 μm mesh sieve. This concentration was selected based on preliminary screening of mechanical and thermal properties of the following compositions: 0.25, 0.4, 0.8, 1.6 and 2.4 wt.%. At 0.25 wt.% we have not noticed any significant effects, and in comparison, between 0.4 wt.% and 0.8 wt.% the latter showed the best performance. Further increases in GO loading required substantially higher sintering temperatures and rendered the powder unsuitable for reuse in subsequent SLS cycles. The samples were obtained on a 3DSystems sPro 60 machine with a scanning speed of 6 m/s, a layer thickness of 100 μm and variable parameters of laser power and chamber temperature. For mechanical properties we produced test samples according to ISO 527-2:2012 [14]. Additionally, porous samples first formed by SLS from untreated PA12 powder were then impregnated with GO by immersion for 10 min followed by drying at 80 °C for 20 min. The impregnation consisted of 3 cycles. For comparison we also produced the same composites from PA12/GO powder by pressing at temperature 205 °C and a pressure of 40 MPa.

Particle size analysis of powders was performed on a Masterizer 2000 (Malvern Panalytical, Malvern, UK) laser analyzer. Powder morphology, surface and fractures of synthesized samples were observed using a Vega 3 Tescan (Tescan, Brno, Czech Republic) scanning microscope (SEM). Mechanical tensile tests were performed on a SHIMADZU AGS-X 100kN (Shimadzu, Kyoto, Japan) universal testing machine. The electrophysical properties of the composites were determined on flat disks of 100 mm diameter and 3 mm thickness using RLC E7-20 (MNIPI, Minsk, Republic of Belarus) and teraohmmeter TOMM-01 (NPP Norma, Samara, Russia). Thermal diffusivity was measured by laser flash method using Netzsch LFA447 (Netzsch Gruppe, Selb, Germany) on 8 by 8 mm and 1 mm thick square samples.

## 3. Results and Discussion

### 3.1. Selective Laser Sintering of Composites

**Figure 1:**
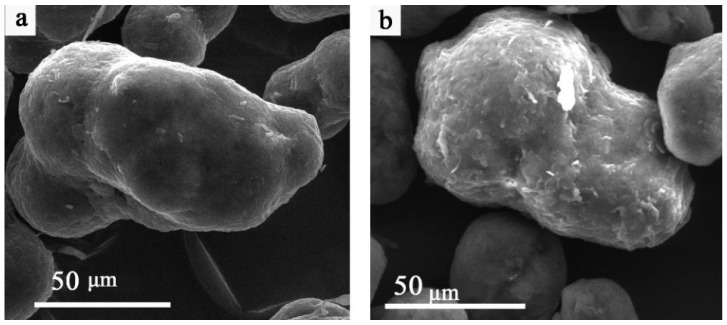
SEM images of pristine polyamide PA12 powder (**a**) and treated with graphene oxide (GO) PA12/GO (**b**).

In selective laser sintering (SLS), numerous parameters—such as the initial powder particle size, layer thickness, laser power, number of passes, chamber temperature, scanning speed, and scan spacing—can significantly influence the quality of the final product. As a first step, the particle size and morphology of pristine PA12 and PA12 following GO treatment were examined using SEM, as presented in Figure 1. The inherently rough surface of PA12 particles (Figure 1a) promotes the adhesion of GO across their surface during the impregnation process.

Occasionally, GO agglomerates up to 15 μm in size are observed on the surface of PA12/GO particles, which can be attributed to the intrinsic tendency of graphene to form folds and clusters. The wet-mixing method employed in this study preserves the original geometry of the PA12 powder particles, as they are not subjected to mechanical or chemical stresses during mixing with the GO dispersion.

Analysis of the particle-size distributions for the PA12 and PA12/GO powders showed average particle diameters of 56.1 ± 1 μm and 57.7 ± 1 μm, respectively. These results indicate that no adjustments to layer thickness, scanning speed, or scan spacing are required for the GO-pretreated powder relative to the pristine material. However, other SLS parameters—such as laser power, chamber temperature, and the number of passes—still require further optimization. Table 1 summarizes the sintering parameters employed during the optimization of the SLS process for PA12/GO composite fabrication. Sintering modes 1 and 2 had previously been optimized for pristine PA12 and were shown to produce parts with the required geometry and a high mechanical performance; therefore, they were selected for initial testing. Mode 1, which uses a lower laser power and chamber temperature together with a single laser pass, yields a higher-porosity structure and was subsequently used in the impregnation methodology.

During SLS processing of the PA12/GO powder under modes 1 and 2, upward curling of the sample corners along the *Z*-axis was observed. This behavior indicates non-uniform shrinkage arising from a temperature gradient between the sintering zone and the surrounding build chamber. This effect is attributed to GO pretreatment, which is known to promote heterogeneous nucleation, increasing the crystallization rate and shifting the crystallization onset to higher temperatures, consistent with heterogeneous nucleation mechanisms [15]. As a result, these two modes were deemed unsuitable for SLS processing of GO-modified PA12 powders.

To improve dimensional accuracy and eliminate corner distortion, three additional sintering modes (modes 3–5 in Table 1) were evaluated by increasing the chamber temperature from 173 °C to 176 °C. Under these conditions, no corner curling or visible shrinkage was observed, which we ascribe to a reduction in the crystallization rate. An additional double laser pass (mode 4) and an increase in laser power to 20 W (mode 5) were also tested to enhance polymer–matrix bonding. While the former resulted in dimensional fidelity fully consistent with the 3D model, the latter caused the formation of surface nodules, indicating excessive polymer melting due to overheating.

### 3.2. Impregnation Method

As an alternative to powder pretreatment, post-sintering impregnation with a GO dispersion was investigated as a route for enhancing the thermal properties of the material. Two types of PA12 scaffolds were fabricated using laser powers of 12 W and 18 W (sintering modes 1 and 2) for subsequent impregnation. X-ray computed tomography (Figure S1) revealed scaffold porosities of 38% and 27%, respectively, with the higher porosity corresponding to the lower laser power. Both scaffold types were impregnated with GO dispersion using the same procedure. Optical microscopy (Figure S2) showed that the lower-porosity scaffold exhibited a penetration depth of approximately 200 μm, whereas the more porous scaffold was almost fully impregnated. Gravimetric analysis, performed by weighing the samples before and after impregnation, indicated GO loadings of 0.8 wt.% for mode 1 and 0.25 wt.% for mode 2.

**Figure 2:**
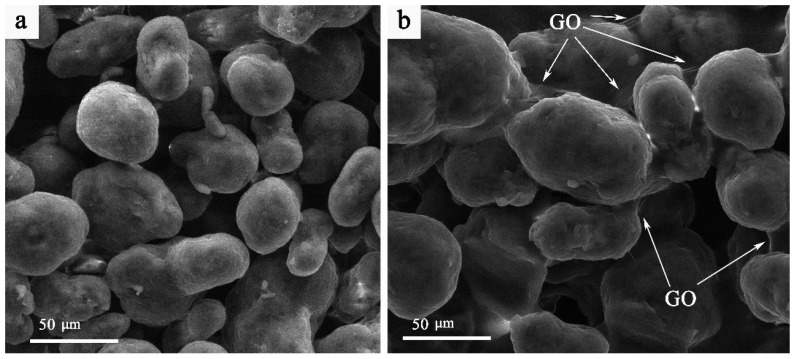
SEM surface imaging of PA12 sample sintered in mode 1 at 12 W before (**a**) and after GO impregnation (**b**).

Figure 2 presents the surface morphology of samples sintered under mode 1 before and after impregnation. Graphene oxide is distributed throughout the pore network and forms agglomerates within the interparticle voids, as indicated by the arrows in Figure 2.

**Figure 3:**
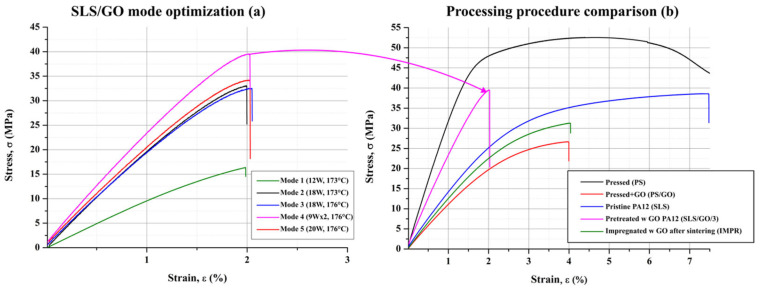
Tensile stress curves of PA12/GO composites obtained in different SLS modes (**a**) and comparison of tensile/strain curves for different production modes: pressed (PS), SLS processed pristine PA12 (SLS), optimized SLS processed PA12 pretreated with GO (SLS/GO/3) and impregnated after the sintering (IMPR) (**b**).

All obtained composites were subjected to tensile testing; the sample designations are summarized in Table 2. The stress–strain curves for PA12 powders pretreated with GO dispersion (SLS/GO) and sintered under different laser modes are shown in Figure 3a. The composite processed using mode 1 exhibited the lowest tensile strength (16.5 MPa). Increasing the laser power (mode 2) resulted in a moderate improvement, yielding a tensile strength of 33 MPa, which was slightly higher than that of the composite produced at an increased chamber temperature (mode 3, 32.5 MPa). We attribute the enhanced strength of the mode 2 sample to the presence of locally hardened regions formed by elevated thermal exposure, particularly at curl locations during printing. Likewise, mode 5—characterized by higher overall energy input—produced a strength of 34.4 MPa, surpassing both mode 2 and mode 3.

The highest ultimate tensile strength was achieved by the composite fabricated under mode 4 (39.5 MPa). This improvement is attributed to the double scanning of each powder layer prior to deposition of the subsequent layer, which facilitates a more gradual energy input, reduces shrinkage and curling, and improves interparticle bonding. Notably, the relative elongation at break varied only marginally across the different sintering modes and remained at 2 ± 0.1%. Based on both mechanical performance and dimensional stability, mode 4 was identified as the optimal sintering regime for PA12/GO composite production, and the corresponding material was designated as SLS/GO/3.

Figure 3b compares the stress–strain curves of pristine PA12 and PA12/GO composites produced by pressing (PS and PS/GO), post-sintering impregnation (IMPR), and pretreatment prior to selective laser sintering (SLS/GO). For pristine PA12, the processing method significantly influenced mechanical performance. Pressed samples (PS) exhibited a higher tensile strength (52.5 MPa) than those produced by laser sintering (SLS, 38.5 MPa). This notable difference is attributed to the intrinsic porosity of SLS-produced parts—up to 30 vol.%—which correspondingly decreases material density from 1.026 g/cm^3^ (PS) to 0.892 g/cm^3^ (SLS).

The PS/GO composite showed a 49% reduction in ultimate strength (26.6 MPa) and a 13-fold decrease in relative elongation (3.9%) compared with pristine PS. The decline in mechanical performance following GO addition is attributed to insufficient filler–matrix interfacial adhesion. The pressing method demonstrated the greatest sensitivity to these interfacial issues, likely due to the formation of GO agglomerates that act as stress concentrators. Among all composites examined, the SLS/GO/3 sample displayed the highest ultimate strength, exhibiting a 2.5% increase relative to pristine SLS-processed PA12. Optimization of the SLS parameters thus enabled the fabrication of composites with enhanced tensile properties. Nevertheless, the incorporation of GO consistently reduced elongation at break, regardless of fabrication method.

**Figure 4:**
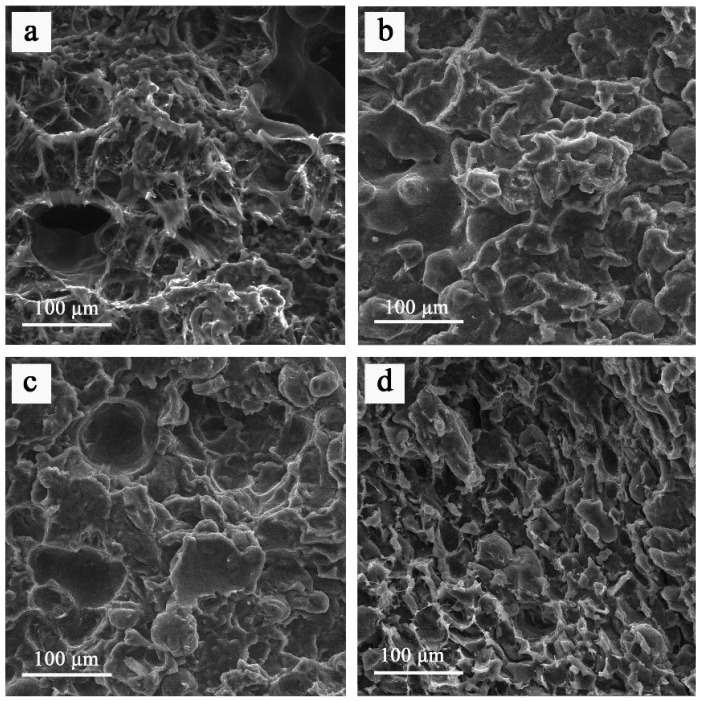
SEM imaging of fracture surfaces for sintered samples SLS (**a**), SLS/GO/1 (**b**), SLS/GO/3 (**c**), PS/GO (**d**).

SEM images of the fracture surfaces (Figure 4) were analyzed for pristine SLS-produced PA12, GO-pretreated PA12 powders processed by SLS (SLS/GO), and composites produced by conventional pressing (PS/GO). The fracture surface of pristine PA12 (Figure 4a) displayed heterogeneous topography and evident breakage of interparticle bonds. The presence of elongated features oriented along the loading direction indicates a predominantly plastic fracture. In contrast, the fracture surfaces of SLS/GO/1, SLS/GO/3, and PS/GO samples (Figure 4b–d) appeared smooth and mirror-like, lacking evidence of material stretching before failure, signifying brittle fracture. These observations are consistent with the reduced elongation at break observed for GO-containing samples.

The fracture morphologies of SLS/GO/1 and SLS/GO/3 resemble that of the pressed material. The absence of distinct particle boundaries indicates a higher degree of particle melting, attributed to improved heat transfer during sintering resulting from the presence of GO on the powder surface.

**Figure 5:**
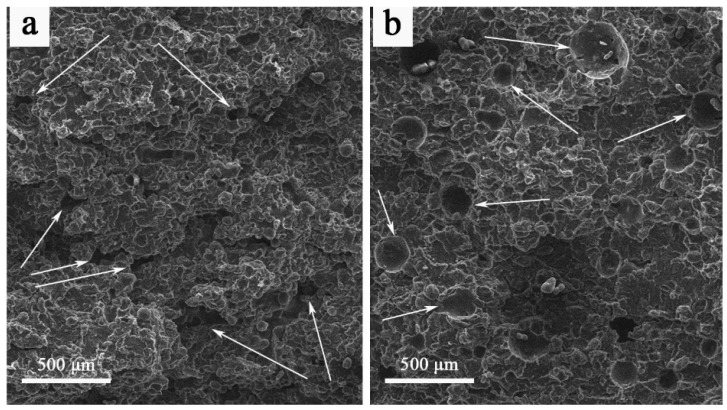
General view of SLS/GO/1, the arrows show irregular shaped pores (**a**) and SLS/GO/3 fractures, the arrows show spherical pores (**b**).

The fracture surface of SLS/GO/1 (Figure 5a) reveals reduced porosity, consistent with increased particle melting and supported by a 3.8% increase in density (0.9275 g/cm^3^) relative to the pristine SLS material (0.892 g/cm^3^). In contrast, the SLS/GO/3 sample (Figure 5b) lacks the typical porous structure of SLS-produced parts and instead contains numerous spherical pores ranging from 30 to 200 μm. The combination of double scanning—providing the highest cumulative thermal input—and enhanced heat transfer from GO pretreatment leads to near-complete melting of the PA12/GO powder and a modified porosity profile.

This optimized sintering mode yields composites with the highest density (0.9315 g/cm^3^; a 4.3% increase relative to pristine SLS) and an improved filler–matrix interconnection, consistent with the superior mechanical properties obtained. However, the extensive melting of powder particles in the absence of applied pressure facilitates the formation of spherical voids within the material, limiting further improvements in mechanical strength and preventing values comparable to those of pressed PA12.

### 3.3. Dielectric Properties

**Figure 6:**
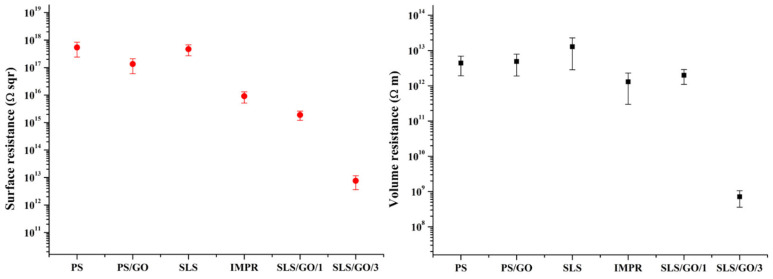
Surface and volume electrical resistance of the composites.

Figure 6 presents the specific bulk and surface electrical resistivity values for pristine sintered PA12 and the corresponding GO-containing composites. For the PS/GO sample, a 9.6% increase in bulk resistivity and a 75% decrease in surface resistivity were observed relative to PS. This behavior is likely associated with the high interfacial resistance between adjacent GO particles, which hinders efficient charge transfer. During pressing, the powder undergoes melting and mechanical compaction, which can disrupt interparticle contacts and promote the formation of GO agglomerates, ultimately preventing the development of a continuous conductive filler network.

In the case of the IMPR composite, surface electrical resistivity decreased from 4.7 × 10^15^ Ω·m (pristine SLS) to 0.78 × 10^15^ Ω·m. A corresponding decrease in bulk resistivity was also observed. These results indicate enhanced electrical performance and confirm the effective penetration and distribution of GO throughout the sample volume when employing post-sintering impregnation. In this approach, GO is deposited along the pore surfaces, forming partially conductive pathways within the finished structure.

The SLS/GO/3 composite exhibited the greatest reduction in both specific volume resistivity (7.6 × 10^8^ Ω·m) and sheet resistivity (7.1 × 10^10^ Ω/sq), indicating the highest electrical conductivity among all tested materials. This composite also displayed the highest dielectric strength (1.58 kV/mm). In laser sintering of PA12/GO powders, the absence of mechanical deformation preserves the initial spatial distribution of GO, enabling a more uniform filler dispersion throughout the sample volume. Moreover, the minimal porosity characteristic of SLS/GO/3 suggests a reduced negative influence of interfacial resistance. The filler-assisted melt diffusion fills the interparticle voids, which establishes additional contact pathways between neighboring powder particles and promotes the formation of conductive networks.

Despite these improvements, the overall electrical resistance of SLS/GO/3 remains high due to the low filler loading, allowing this composite to still be classified as an electrical insulator.

### 3.4. Thermal Conductivity

**Figure 7:**
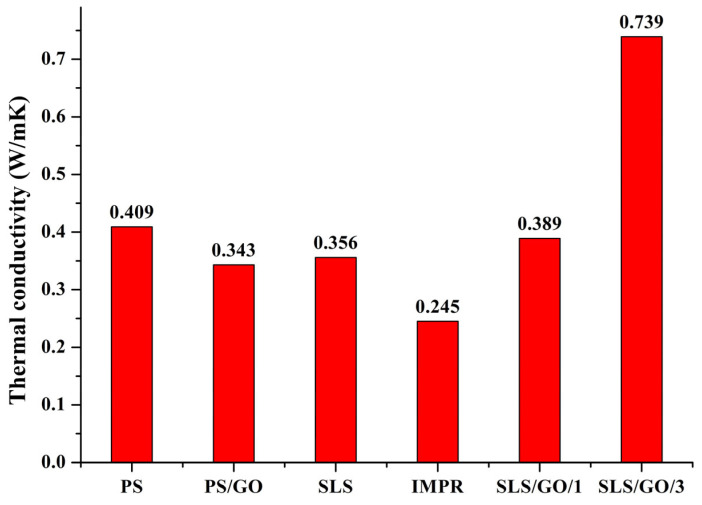
Thermal conductivity of pristine PA12 produced by pressing and SLS methods compared to P12/GO composites that were obtained by GO dispersion pretreatment of PA12 powder, followed by SLS (SLS/GO) and post-sintering impregnation of polymer matrix by GO (IMPR).

The incorporation of GO into polymer matrices can enhance thermal conductivity by enabling the formation of a three-dimensional conductive network through continuous contact between filler particles. We consider two mechanisms of thermal conductivity in solids: electron and phonon heat transfer. Due to the high porosity of SLS-produced PA12, the phonon transfer is impaired mainly because of phonon scattering on defects and poor contact between the particles. In pristine PA12, the electron heat transfer mechanism is negligible due to very high electrical resistance. Optimization of SLS/GO composites with pre-sintering impregnation has allowed us to increase thermal conductivity of both mechanisms simultaneously. First, we obtained significantly higher Young’s modulus in comparison to pristine SLS-produced PA12, due to better particle contacts, which was also confirmed by SEM imaging with brittle fractures of the surface. Second, much lower volume and surface electrical resistance was achieved, dropping 3 to 5 orders of magnitude in optimized SLS/GO-produced composites in comparison to SLS-produced or pressed materials. Although the composite is unlikely to possess fully continuous electrical pathways sufficient for dominant electron-mediated heat transfer (due to the still high resistance of GOhms), a combined contribution from phonon transport and limited electron-assisted heat transfer may account for the observed increase in thermal conductivity. The thermal conductivity values obtained for the composites are presented in Figure 7.

The thermal conductivity of PS/GO was 0.343 W·m^−1^·K^−1^, which is 16% lower than that of pristine PS (0.409 W·m^−1^·K^−1^). This reduction is attributed to interfacial thermal resistance at the filler–matrix interface, which promotes phonon scattering and impedes heat flow. The IMPR composite produced by post-sintering impregnation exhibited an even more pronounced decrease in thermal conductivity—0.245 W·m^−1^·K^−1^, a 31% reduction relative to pristine SLS material (0.356 W·m^−1^·K^−1^). This behavior results from the continuous pore network inherent to the impregnated structure: although intended to facilitate the formation of interconnected filler pathways, the pores introduce dominant thermal barriers that outweigh the conductive contribution of GO.

As noted earlier, the SLS/GO/1 and SLS/GO/3 composites exhibit greater particle fusion and minimal porosity, which improve matrix–filler interfacial contact and reduce thermal boundary resistance. The simultaneous suppression of structural defects and the more uniform distribution of GO—leading to the formation of a continuous three-dimensional filler network during laser sintering—resulted in the highest thermal conductivities among all tested materials: 0.389 W·m^−1^·K^−1^ for SLS/GO/1 and 0.739 W·m^−1^·K^−1^ for SLS/GO/3.

**Figure 8:**
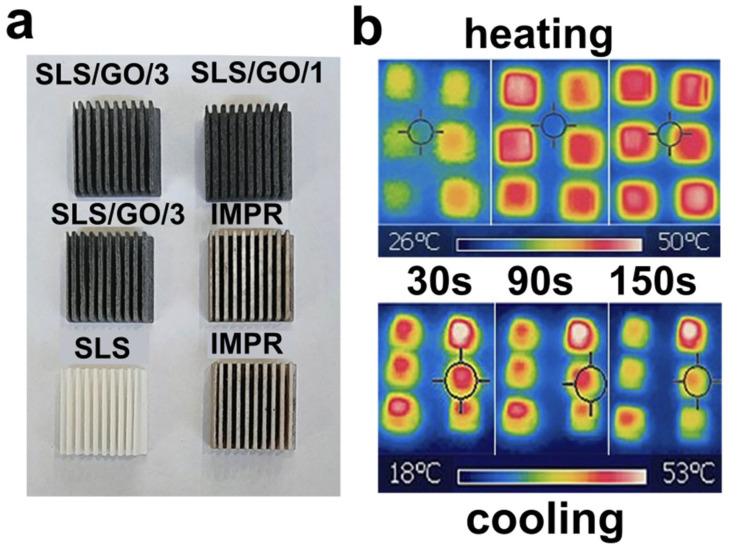
Photograph of the radiators formed from pristine PA12 (SLS), treated with GO (SLS/GO) and impregnated (IMPR) by the dispersion after the sintering (**a**), and thermal imaging of the same pieces during heating and cooling sampled after 30, 90, and 150 s (**b**).

To evaluate performance under conditions closer to practical application, prototype radiators corresponding to SLS/GO/3, SLS/GO/1, IMPR, and pristine SLS were fabricated. Figure 8 shows photographs of the prototypes as well as thermal images during heating and cooling from 25 °C to 53 °C. As observed, the SLS/GO/3 prototypes heat and cool at the fastest rates, consistent with their superior thermal conductivity. The IMPR prototypes also exhibit improved heating and cooling kinetics relative to pristine SLS, attributable to the comparatively higher GO content achieved during impregnation; in these thin-walled structures (0.7 mm), the GO-enriched surface layers constitute a significant fraction of the total volume. The SLS/GO/1 prototype demonstrated slower thermal response, which we attribute to deviations from the intended geometry. These deviations likely arise from additional powder adhesion during printing caused by localized temperature increases in the narrow features of the part. We also summarize all the data in Table 3 for easier comparison between all the samples and preparation techniques used.

These findings highlight the importance of simultaneously considering both the targeted functional properties and the geometric characteristics of the final component when selecting the fabrication method and sintering parameters for GO-reinforced polymer composites.

## 4. Conclusions

In this study, polyamide-12–based composites containing 0.8 wt.% GO were fabricated using pressing, post-sintering impregnation, and selective laser sintering (SLS). The use of a double-scanning strategy in the SLS process significantly reduced the porosity typically associated with laser-sintered materials and resulted in a 2.5% increase in tensile strength compared with pristine PA12. Furthermore, the optimized SLS/GO composites exhibited a thermal conductivity of 0.74 W·m^−1^·K^−1^, attributable to improved matrix–filler interfacial interactions and the formation of a more continuous heat-transfer network.

The combination of enhanced thermal conductivity at low filler loading, high electrical resistivity, and satisfactory mechanical performance highlights the potential of these composites for applications in thermal management, lightweight conductive elements, and other advanced engineering systems. The advantages of the SLS process—particularly its tunability, geometric flexibility, and suitability for complex architectures—further expand the opportunities for integrating GO-modified PA12 composites into diverse scientific and technological domains.

## Figures and Tables

**Table 1:** SLS mode parameters used during the process optimization for SLS/GO composites.

Mode	Laser Power, W	Chamber Temperature, °C	Number of Laser Passes
**1**	12	173	1
**2**	18	173	1
**3**	18	176	1
**4**	9	176	2
**5**	20	176	1

**Table 2:** Designation of composite samples.

Composite Designation	Powder	Method	
**PS**	PA12	Pressing	
**PS/GO**	PA12/GO		
**SLS**	PA12	Selective laser sintering	Mode 2
**SLS/GO/1**	PA12/GO		Mode 3
**SLS/GO/2**	PA12/GO		Mode 1
**SLS/GO/3**	PA12/GO		Mode 4
**IMPR**	PA12	Impregnation after selective laser sintering	

**Table 3:** Mechanical, electrical, and thermal properties of the composites and pristine PA12.

	PS	PS/GO	SLS	SLS/GO/1	SLS/GO/3	IMPR
Tensile strength, MPa	52.5 ± 0.3	26.6 ± 0.3	38.5 ± 0.3	32.5 ± 0.3	39.5 ± 0.3	31.2 ± 0.3
Elongation at break, %	54.5 ± 0.1	3.9 ± 0.1	7.4 ± 0.1	1.9 ± 0.1	2.0 ± 0.1	4.1 ± 0.1
Young’s Modulus, GPa	3.1	1.1	1.4	1.97	2.24	1.26
Surface resistivity, Ω·m	(5.4 ± 3) × 10^15^	(1.35 ± 0.75) × 10^15^	(4.7 ± 2) × 10^15^	(1.9 ± 0.7) × 10^13^	(7.6 ± 4) × 10^10^	(9.1 ± 4) × 10^13^
Volume resistivity, Ω·m	(4.4 ± 2.5) × 10^9^	(4.9 ± 3) × 10^9^	(1.3 ± 1) × 10^10^	(2 ± 0.9) × 10^9^	(7.1 ± 3.5) × 10^5^	(1.3 ± 1) × 10^9^
Thermal conductivity, W·m^−1^·K^−1^	0.409	0.343	0.356	0.389	0.739	0.245
Density, g/cm^3^	1.026	0.982	0.892	0.9275	0.9315	0.71
Intrinsic porosity, %	-	-	27 ± 3	25 ± 3	24 ± 3	38 ± 3

## Data Availability

The original contributions presented in this study are included in the article/Supplementary Material. Further inquiries can be directed to the corresponding authors.

## Supplementary Materials

The following supporting information can be downloaded at: https://www.mdpi.com/article/10.3390/nano16030170/s1, Figure S1. X-ray computed tomography porosity imaging of sintered PA12 samples obtained at 12 W (left) and 18 W of power (right), with later showing much lower porosity. Figure S2. Microphotographs of sintered PA12 samples obtained at 12 and 18 W of power (mode 1 and 2) showing higher penetration of GO inside the material obtained at 12 W and only 200 um depth of impregnation for the one obtained at 18 W. Figure S3. AFM image of GO flakes drop-casted and dried on Si/SiO_2_ surface showing flakes 4–6 um in diameter and 1 nm in height. Figure S4. EDX spectrum of GO material dried on top of Si/SiO_2_ substrate showing C, O and S bands (the main peak is Si from the substrate, which is excluded from the calculation). Sulfur residue is due to usage of H_2_SO_4_ during the synthesis. Figure S5. Raman spectrum of GO sample showing broad D (1345 cm^−1^) and G (1601 cm^−1^) band, which are characteristic in this material.
