# Supplementary material for: Graphene Oxide (GO) Impregnation of Polyamide-Based Composites Enhances Thermal Conductivity After Selective Laser Sintering
Source: Nanomaterials (Basel). 2026 Jan 27;16(3):170. doi: 10.3390/nano16030170 (PMC12899130; doi:10.3390/nano16030170)
Supplement: Supplementary file 1 [file nanomaterials-16-00170-s001.zip › nanomaterials-4062033-supplementary.pdf]

Graphene Oxide (GO) Impregnation of Polyamide-Based Composites Enhances Thermal Conductivity After Selective Laser Sintering

Viktoria A. Koshlakova [1], Andrey A. Stepashkin [2], Valter Maurino [3,4,*] and Dmitry S. Muratov [3,4,*]

[1] Department of Functional Nanosystems and High-Temperature Materials, National University of Science and Technology "MISIS", 119049 Moscow, Russia; vikakoshlakova@yandex.ru
[2] Center of Composite Materials, National University of Science and Technology "MISIS", 119049 Moscow, Russia; a.stepashkin@yandex.ru
[3] Department of Chemistry, University of Turin, 10125 Turin, Italy
[4] UNITO-ITT JointLab, via Quarello 15/a, 10135 Turin, Italy
* Correspondence: valter.maurino@unito.it (V.M.); dmitry.muratov@unito.it (D.S.M.)

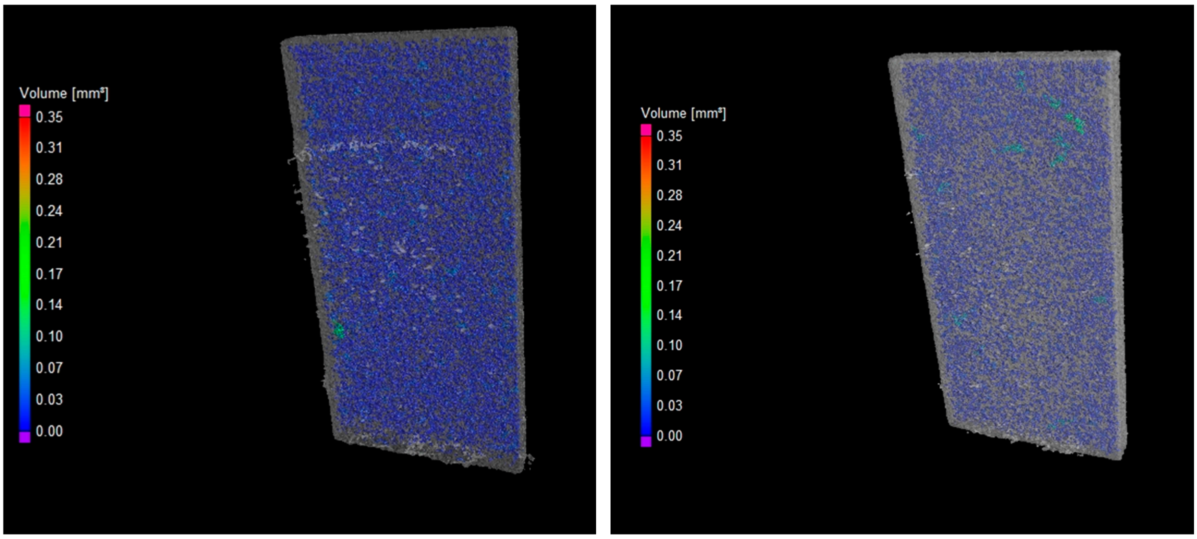

Figure S1. X-Ray computed tomography porosity imaging of sintered PA12 samples obtained at 12 W (left) and 18 W of power (right), with later showing much lower porosity

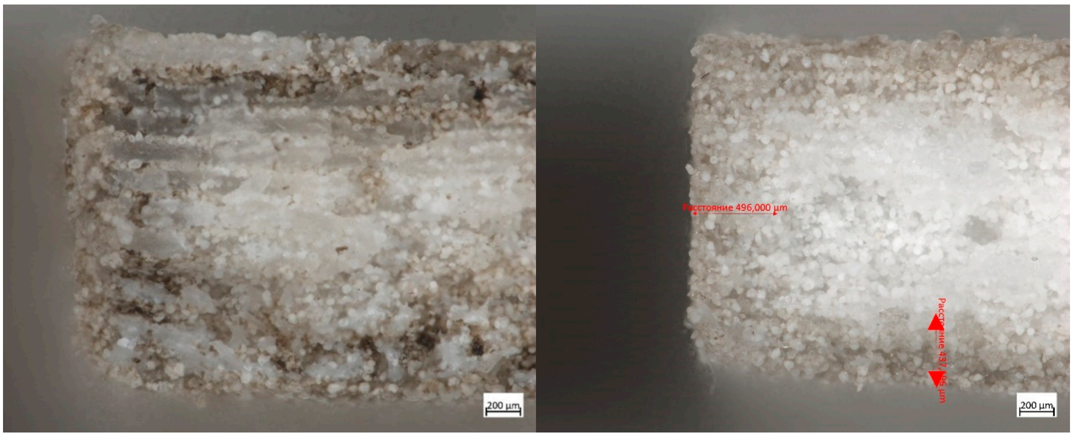

Figure S2. Microphotographs of sintered PA12 samples obtained at 12 and 18 W of power (mode 1 and 2) showing higher penetration of GO inside the material obtained at 12 W and only 200 um depth of impregnation for the one obtained at 18 W

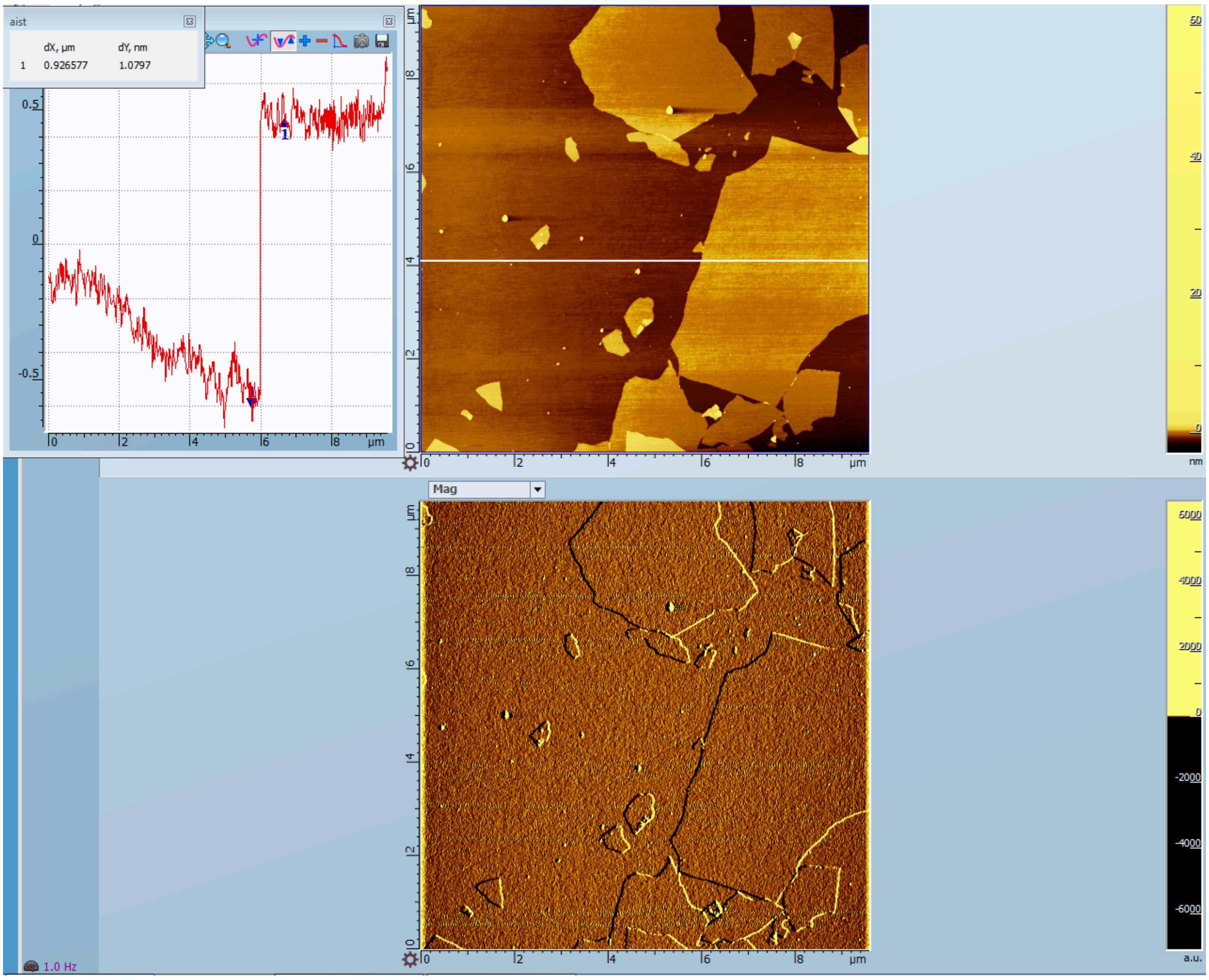

Figure S3. AFM image of GO flakes drop-casted and dried on Si/SiO_2 surface showing flakes 4-6 um in diameter and 1 nm in height

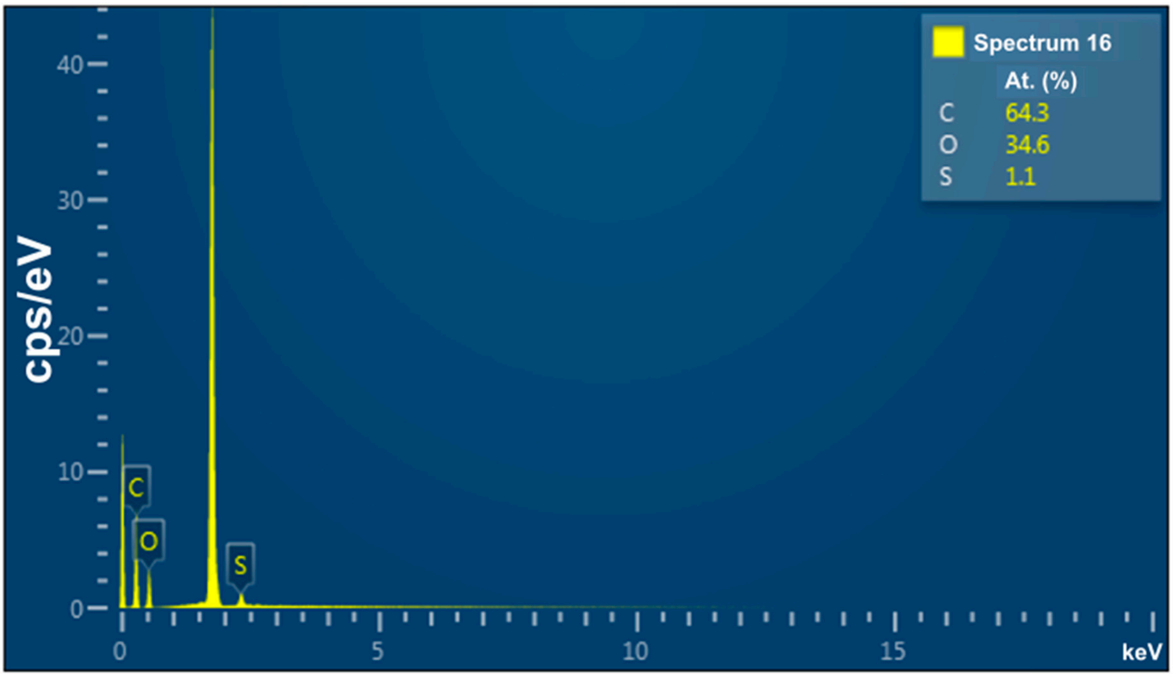

Figure S4. EDX spectrum of GO material dried on top of Si/SiO_2 substrate showing C, O and S bands (the main peak is Si from the substrate, which is excluded from the calculation). Sulfur residue is due to usage of H_2SO_4 during the synthesis

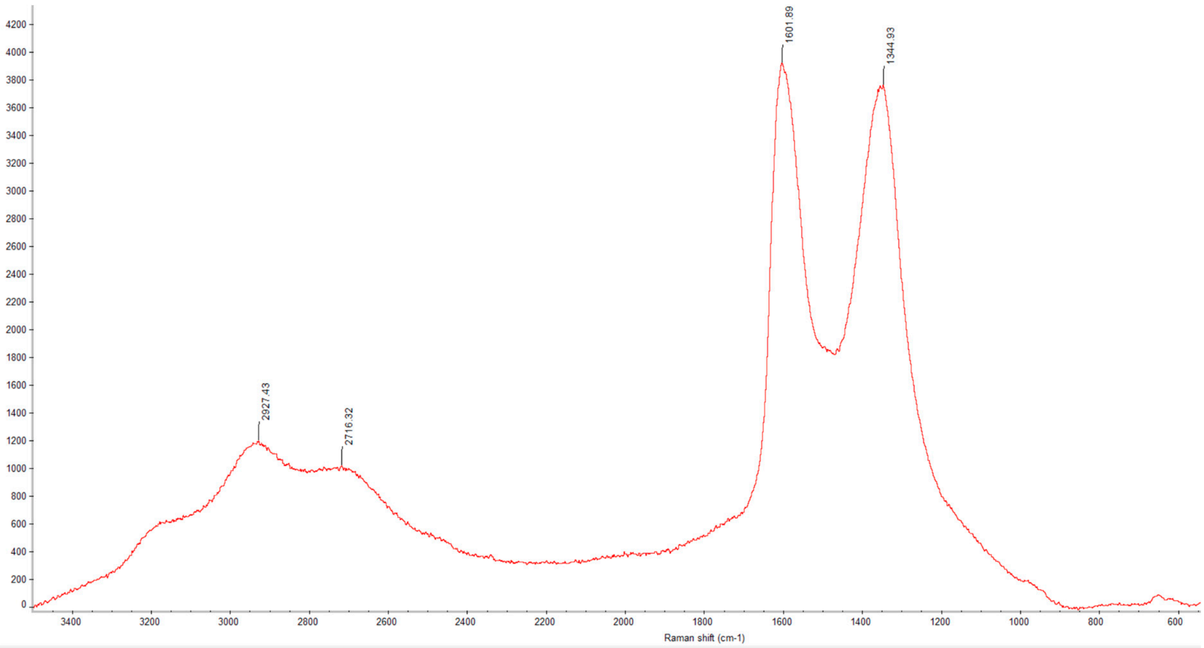

Figure S5. Raman spectrum of GO sample showing broad D (1345 cm^{-1}) and G (1601 cm^{-1}) band, which are characteristic in this material
